# Limit of the electrostatic doping in two-dimensional electron gases of LaXO_3_(X = Al, Ti)/SrTiO_3_

**DOI:** 10.1038/srep06788

**Published:** 2014-10-27

**Authors:** J. Biscaras, S. Hurand, C. Feuillet-Palma, A. Rastogi, R. C. Budhani, N. Reyren, E. Lesne, J. Lesueur, N. Bergeal

**Affiliations:** 1 LPEM- UMR8213/CNRS - ESPCI ParisTech - UPMC, 10 rue Vauquelin - 75005 Paris, France; 2 Condensed Matter - Low Dimensional Systems Laboratory, Department of Physics, Indian Institute of Technology Kanpur, Kanpur 208016, India; 3 National Physical Laboratory, New Delhi - 110012, India and; 4 Unité Mixte de Physique CNRS-Thales, 1 Av. A. Fresnel, 91767 Palaiseau, France

## Abstract

In LaTiO_3_/SrTiO_3_ and LaAlO_3_/SrTiO_3_ heterostructures, the bending of the SrTiO_3_ conduction band at the interface forms a quantum well that contains a superconducting two-dimensional electron gas (2-DEG). Its carrier density and electronic properties, such as superconductivity and Rashba spin-orbit coupling can be controlled by electrostatic gating. In this article we show that the Fermi energy lies intrinsically near the top of the quantum well. Beyond a filling threshold, electrons added by electrostatic gating escape from the well, hence limiting the possibility to reach a highly-doped regime. This leads to an irreversible doping regime where all the electronic properties of the 2-DEG, such as its resistivity and its superconducting transition temperature, saturate. The escape mechanism can be described by the simple analytical model we propose.

Two-dimensional electron gases (2-DEGs) at LaAlO_3_/SrTiO_3_ and LaTiO_3_/SrTiO_3_ oxide interfaces^1^ have attracted much attention since their electronic properties display a very rich physics with various electronic orders such as superconductivity^2^,^3^,^4^ and magnetism^5^,^6^,^7^,^8^. In these structures, the 2-DEG is confined in an interfacial quantum well that typically extends on the order of 10 nm into the SrTiO_3_ substrate at low temperature^2^,^4^,^9^,^10^,^11^. Applying a back-gate voltage enables to control electrostatically the filling of the well and thus modulate the 2-DEG electronic properties^12^,^13^,^14^. This exciting feature opens new avenues for studying the electronic orders and quantum phase transitions^15^ in theses structures, as well as for developing oxide-based electronics that could make use of them^16^,^17^,^18^,^19^. Of particular interest, it was shown that adding electron to the well, increases continuously the electronic mobility and the strength of the Rashba spin-orbit coupling^14^,^20^,^21^. Also of interest, is the fact that the superconducting transition temperature of the 2-DEG exhibits a dome-like shape with a maximum T*_c_* of 200–300 mK at optimal doping^13^,^14^. Because of these remarkable properties, the highly-doped regime must be explored in further detail. However, the overdoped side of the dome seems particularly difficult to study because of unexplained saturation and hysteresis of the physical properties^13^,^14^,^20^. As of yet, this region is not fully understood. These observations raise a fundamental question: how much one can electrostatically dope the 2-DEG at oxides interfaces?

Although several theoretical descriptions of LaXO_3_(X = Al, Ti)/SrTiO_3_ interfaces have been proposed^22^,^23^,^24^,^25^,^26^,^27^, the exact interfacial band structure remains controversial. Regardless of the calculation method and for the sake of clarity, in the present report we will consider a simple generalized situation where the bending of the SrTiO_3_ conduction band defines a quantum well that accommodates discrete electronic sub-bands. To illustrate this point, we show in the Figure 1 the type of result that can be obtained by using a semiconductor approach in which we solve self-consistent Schrödinger-Poisson equations^14^,^28^. In this example, six sub-bands are filled and the higher energy one extends within 5 nm in the SrTiO_3_^10^,^11^; this situation corresponds to a carrier density of approximately 7 × 10^13^ e^−^/cm^2^, typical of values found in the literature. The effect of a back gate voltage on the interface is twofold: (i) it adds electrons to the well as the gate voltage increases (Δ*V*_G_ > 0) and removes electrons from the well as the gate voltage decreases (Δ*V*_G_ < 0), (ii) it controls the shape of the upper part of the well by tilting the conduction band profile in the substrate. Figure 2 shows illustrations of quantum well energy profiles that describe these different situations. As long as the Fermi level remains deep in the well, electrostatic gating can reversibly empty and fill the well as in the well-known semiconductors quantum wells. However, when the Fermi level rises to the top of the well, we no longer expect the doping to be possible. In this article we show that in LaXO_3_(X = Al, Ti)/SrTiO_3_ heterostructures, the Fermi energy lies intrinsically near the top of the well and that, beyond a filling threshold, additional electrons irreversibly escape from the well, hence limiting the possibility to reach a highly-doped regime. This behaviour can be described by a simple analytical model based on a thermal activated mechanism.

## Results

### First positive polarization

**Figure 2:**
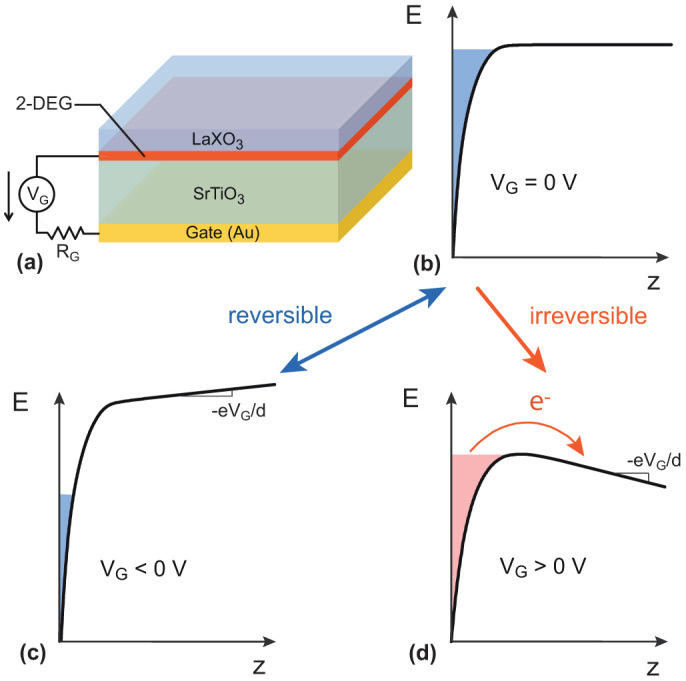
(a) Schematic of an LaXO_3_(X = Al, Ti)/SrTiO_3_ sample with a metallic back gate. (b,c,d) Illustration of filling the quantum well for different *V*_G_ regimes. Starting from *V*_G_ = 0, applying a negative gate voltages *V*_G_ < 0 reversibly empties and fills the well. In contrast, applying the first positive polarisation (*V*_G_ > 0), causes electrons to irreversibly escape from the well. The slope −*eV*_G_/*d* (where *d* = 500 *μ*m is the thickness of the SrTiO_3_ substrate) of the conduction band is indicated.

**Figure 3:**
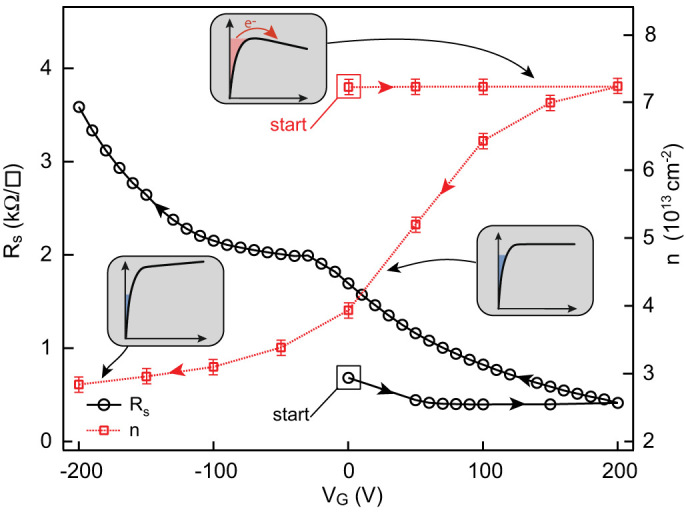
Resistance of the 2-DEG (left axis) and carrier density *n* (right axis) as functions of gate voltage measured for the first forward sweep (first positive polarization) and reverse sweep. The starting points and the directions of the sweeps are indicated by enclosing boxes and arrows respectively.

In these experiments we used LaTiO_3_ and LaAlO_3_ epitaxial layers grown on TiO_2_-terminated SrTiO_3_ single crystals by Pulsed Laser Deposition as described in the Methods section. The samples had typical dimensions of 1 × 2 mm^2^ and each had a metallic back-gate deposited at the rear of the 0.5 mm thick SrTiO_3_ substrate. Before cooling, samples are kept in the dark for more than twelve hours to suppress any photoconductive effects. We studied the transport properties of LaXO_3_(X = Al, Ti)/SrTiO_3_ heterostructures at 4.2 K while applying a positive gate voltage for the first time, referred to as the first positive polarization. We first focus on the response of the LaTiO_3_/SrTiO_3_ sample shown in Figure 3. In this sample, increasing the gate voltage causes the resistance of the 2-DEG, after decreasing slightly, to saturate quickly and become independent of gate voltage. This behaviour is unexpected because, according to electrostatic laws, more electrons are added to the 2-DEG and therefore the resistance should decrease. This first positive polarization is irreversible: when the gate voltage is decreased from its maximum at 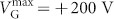, the reverse resistance curve deviates from the first forward curve. The carrier density *n* was extracted from a two-carrier analysis of the Hall effect at high magnetic field (45 T) as described in reference [14] and Supplementary material. It was found to be constant during the first positive polarization, a behaviour consistent with the saturation of the resistance. Similar to the resistance curve, when the gate voltage is decreased after being increased to 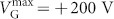, the reverse carrier density curve does not follow the first forward curve. These two behaviours are in agreement with the irreversible situation described in Figure 2d. After the heterostructure is initially cooled, its Fermi level lies very close to the top of the well. Increasing the gate voltage adds electrons at the interface that quickly fills the highest energy sub-bands at the top of the well. After a certain time, these electrons eventually escape into the conduction band of the SrTiO_3_ substrate, causing the saturation of the carrier density and the resistance of the 2-DEG. Note that this saturation is not associated with an increase of the gate current, suggesting that charges are trapped in the system.

**Figure 4:**
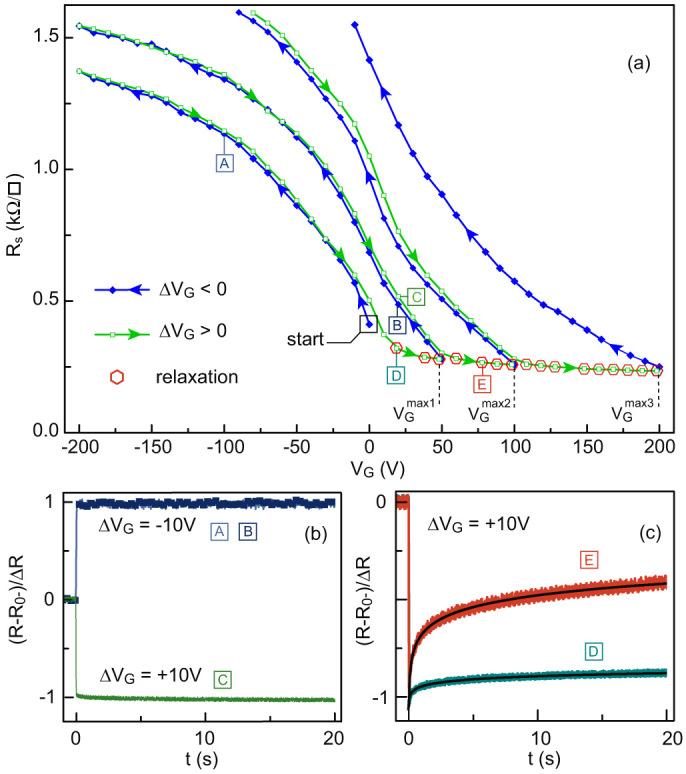
Resistance of the LaTiO_3_/SrTiO_3_ sample measured for several forward sweeps (Δ*V*_G_ > 0) and reverse sweeps (Δ*V*_G_ < 0) with a starting *V*_G_ of 0 V (“start” point). We first sweep the *V*_G_ to −200 V and then increase it to a maximum value 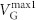 before sweeping it back to −200 V. We repeated this operation with increasing gate voltage maximum of 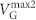 and 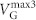. Red markers indicate the region in which we observed relaxation. (b) Normalized resistance over time measured as a function of time after a Δ*V*_G_ = ±10 V step in points labelled “A”, “B” and “C” on the *R*(*V*_G_) curves of panel (a). (c) Normalised resistance over time after a Δ*V*_G_ = +10 V step at points labelled “D” and “E” on the *R*(*V*_G_) curves in (a). The resistance relaxation is fitted to a logarithm with form *α* + *β* log(*t*) (solid black lines).

Beyond our initial experiments, we studied the irreversibility of the first positive polarization in further detail. After cooling a LaTiO_3_/SrTiO_3_ heterostructure to 4.2 K, we measured its sheet resistance as a function of gate voltage by using different polarization procedures (Fig. 4a). Different from our previous measurements, in this experiment we applied a negative first polarization down to 
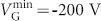
. Doing so increased the resistivity – an expected behaviour, as electrons are removed from the 2-DEG – and we observed no saturation. As the voltage returns to *V*_G_ = 0 V, the forward resistance curve appears to match the first reverse curve. However, when the gate voltage is further increased to the value 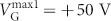 and then decreased back to *V*_G_ = −200 V, the reverse resistance curve deviates from the first forward curve. This new curve is fully reversible as long as the gate voltage is not increased above 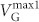. We replicated this pattern with increasing maximum gate voltages of *V*_G_ (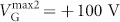, and 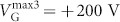).

**Figure 5:**
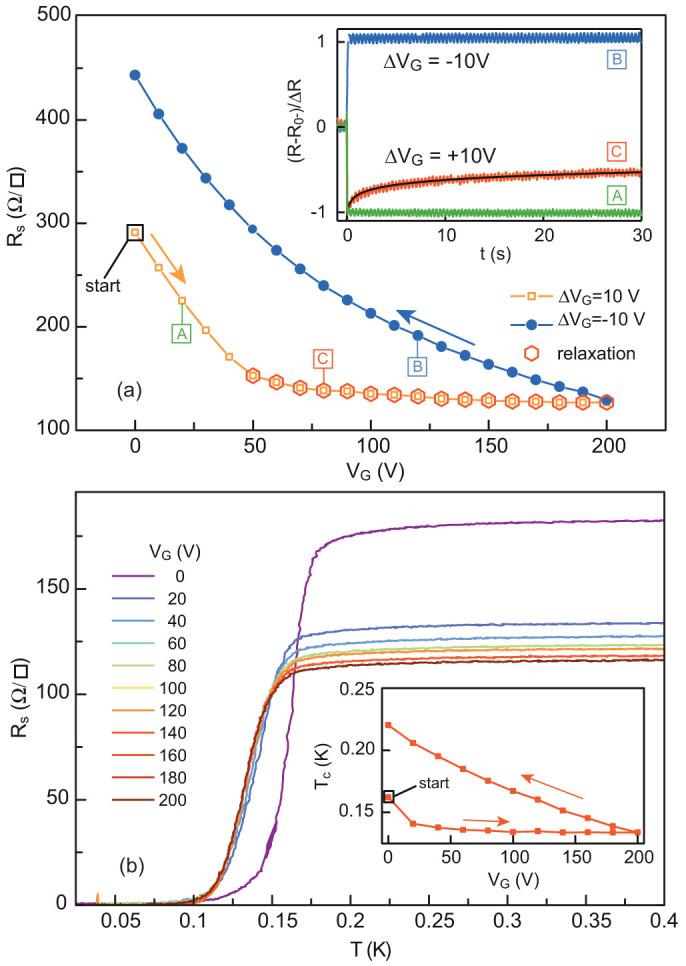
(a) Sheet resistance of the LaAlO_3_/SrTiO_3_ sample during the first positive polarization at 4.2 K. Inset: Normalised resistance over time after a Δ*V*_G_ = ±10 V step for points labelled “A”, “B” and “C” on the *R*(*V*_G_) curves in the main panel. The relaxation of the resistance is fitted to a logarithm with the form *α* + *β* log(*t*). (b) Sheet resistance of the LaAlO_3_/SrTiO_3_ sample as a function of temperature for different *V*_G_ values during the first positive polarization, showing the saturation of *T_c_*. Inset: Hysteresis of *T_c_* as a function of *V*_G_ for the first positive polarization.

These results show that the well can be emptied (Δ*V*_G_ < 0) and filled (Δ*V*_G_ > 0) reversibly as long as the gate voltage is not increased beyond a critical value corresponding to the maximum value 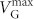 previously applied to the metallic gate, a situation in which the Fermi level reaches the top of the well. Beyond this maximum value, we expect electrons to escape irreversibly from the 2-DEG into the SrTiO_3_ substrate. We performed the same measurements on an LaAlO_3_/SrTiO_3_ sample and we observed similar results (Fig. 5a). For this sample, the irreversible regime is reached at a gate voltage of 50 V indicating that the Fermi level after the initial cooling was slightly below the top of the well. As a consequence, at lower temperatures, the superconducting transition temperature saturates beyond this gate value (Fig. 5b and inset). To suppress the undesirable hysteresis effects, a first positive polarisation can be used as a forming process of the quantum well prior to other measurements^13^,^14^,^20^. However, in any case, the heavy doping of the 2-DEG will always be limited because the Fermi level cannot exceed the top of the quantum well.

### Time-dependent measurement

We performed time-dependent resistivity measurements to assess how the 2-DEG responds to gate voltage steps of Δ*V*_G_ = ±10 V. The expected corresponding modification of the carrier density is Δ*n* = *C_a_*Δ*V*_G_/*e* where *C_a_* is the capacitance per unit of area of the SrTiO_3_ substrate. Representative results of these measurements are shown in Figure 4b and 4c for different filling situations, labelled “A”, “B”, “C”, “D” and “E” in Figure 4a. In the reversible regime, the resistance shows clears Δ*R* jumps before reaching a stable value (Fig. 4b). As expected, Δ*R* is positive when electrons are removed (Δ*V*_G_ = −10 V, labels “A” and “B”) and negative when electrons are added (Δ*V*_G_ = +10 V, label “C”). In contrast, after applying a voltage step of Δ*V*_G_ = +10 V in the irreversible regime, the initial negative jump is followed by a slow increase of the resistance (Fig. 4c, labels “D” and “E”). We interpret this behaviour as the sign that electrons added to the 2-DEG by the gate-voltage step, eventually escaped from the well. By a first approximation, the resistance relaxation follows a logarithmic time dependence with the form *α* + *β* log(*t*). This relaxation must not be confused with the charging time of the capacitor *R*_G_*C_a_A* (A is the area of the sample) which is always present at a much shorter time scale (see Supplementary Information).

## Model and Discussion

**Figure 6:**
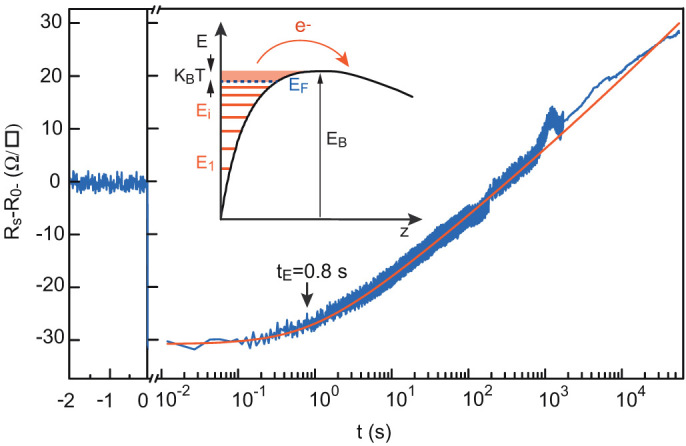
Resistance over time of the LaTiO_3_/SrTiO_3_ sample after a Δ*V*_G_ = +10 V step at t = 0, fitted by equation (6). The escape time *t*_E_ is 0.8 ± 0.1 s. Inset: Schematic of the situation considered to model the thermal escape of the electrons from the well.

To analyze the relaxation in the irreversible regime we propose a model that describes the dynamics of electrons escaping from the well. We consider a 2D quantum well at the interface with an infinite barrier on the LaXO_3_(X = Al, Ti) side and a barrier of finite height *E_B_* on the SrTiO_3_ side (Inset of Fig. 6). A number *n_L_* of 2D parabolic sub-bands with energy *E_i_* (*i* = 1,…,*n_L_*) and density of states 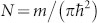 are filled. We assume that at a temperature *T*, electrons at the Fermi level *E*_F_ can jump over the barrier with first order kinetics: 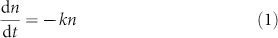
where *n* is the carrier density of the 2-DEG and *k* is the kinetic factor. This latter follows an Arrhenius law: 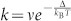 where the activation energy is Δ = *E_B_* − *E*_F_, and *ν* is a characteristic frequency factor. In two dimensions, the electron density is given by 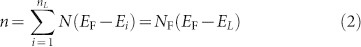
where *N*_F_ = *n_L_N* is the total density of states at the Fermi energy and 
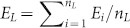
. This situation is formally equivalent to the one with a single band of energy *E_L_* and a density of state *N*_F_. For a small variation of *n*, the temporal evolution of the Fermi energy is 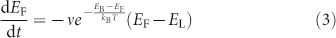
At low temperature (*k_B_T* ≪ *E*_F_ − *E_L_*), a good approximate solution to equation (3) is 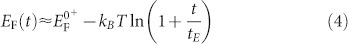
where 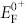 is the Fermi level at *t* = 0^+^ (immediately after the voltage step, neglecting the short charging time *R*_G_*C_a_A*) and *t_E_* is the characteristic escape time given by 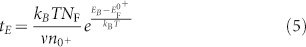
where 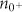 is the carrier density at *t* = 0^+^. Therefore, the Fermi level is constant for *t* < *t_E_*, after which it decreases logarithmically. From (2) and (4) we can obtain the temporal dynamics of the 2-DEG Drude resistivity as 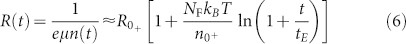
where 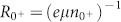 is the resistivity at *t* = 0^+^. In Figure 6 we show the resistance relaxation after a Δ*V_G_* = +10 V step; this relaxation agrees very well with equation (6) over more than six decades of time (10 ms to 14 hours). A direct consequence of the logarithmic relaxation is the absence of an asymptotic value. However, on a linear scale the resistance changes very slowly after a few minutes, which can give a false impression of saturation. We emphasise here that the peculiar form of relaxation given by Eq. 6 is specific to the case of a well that empties itself and cannot describe other thermally activated mechanisms.

**Figure 7:**
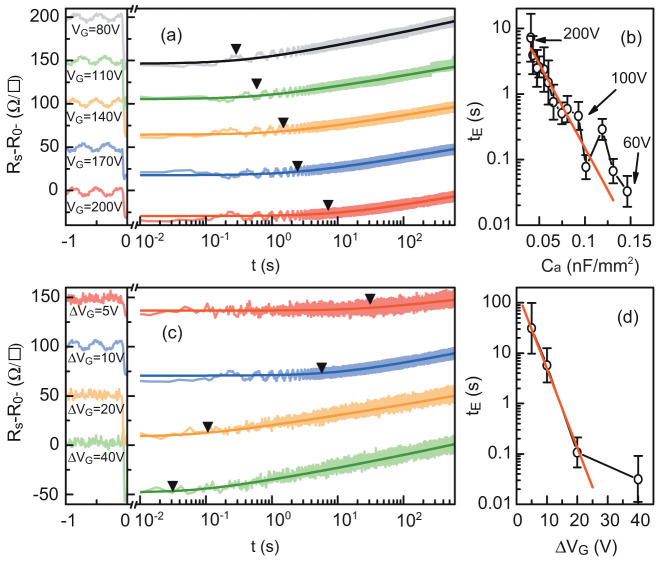
(a) Resistance over time of the LaTiO_3_/SrTiO_3_ sample, measured at 4.2 K for different gate voltages after a Δ*V_G_* = +10 V step, fitted by equation (6). Arrows indicate *t_E_* values extracted from the fits. (b) Logarithm of *t_E_*, plotted as a function of *C_a_* and fitted with equation (7) (red line). (c) Resistance over time, measured at 4.2 K for different steps Δ*V*_G_, and fitted with equation (6). Arrows indicate *t_E_* values extracted from the fits. (d) Logarithm of *t_E_* plotted as a function of Δ*V*_G_ and fitted with equation (7).

To validate this model, we systematically studied how the relaxation depends on the polarization parameters. In particular, we measured the resistance relaxation after a Δ*V_G_* = +10 V step at different *V*_G_ values (Fig. 7a), and for different steps of Δ*V*_G_ = 5, 10, 20 and 40 V (Fig. 7c). The experimental data from both experiments agree with the theoretical equation (6), confirming that the model describes the phenomena we observed very well. We also observed the same agreement between experimental data and theoretical expectations for the LaAlO_3_/SrTiO_3_ sample (Supplementary Material). To understand how the escape time depends on *V*_G_ and Δ*V_G_*, we can express equation (5) as 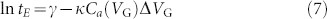
where *γ* and *κ* are constants whose expressions can be found in the Supplementary Material. Because the dielectric constant of SrTiO_3_ is electric-field-dependent, the capacitance *C_a_* changes with gate voltage^29^. Therefore, the number of charges added by a constant voltage step Δ*V*_G_ depends on the absolute value of the gate voltage. Figure 7b shows the linear variation of ln *t_E_* as a function of *C_a_* as expected from equation (7). We also found ln *t_E_* to vary linearly with Δ*V_G_* for small gate voltage step Δ*V*_G_ (figure 7d). For larger steps, the electrons are injected very high in the well complicating our determination of the short *t_E_* values.

**Figure 8:**
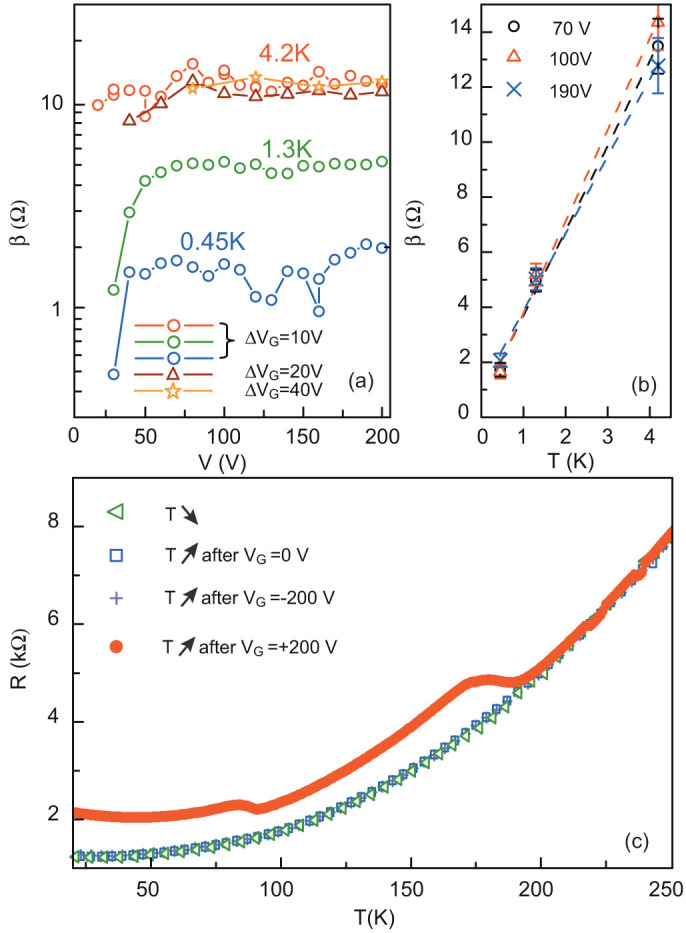
(a) Parameter *β* of the logarithmic fit of the relaxation curves, as a function of gate voltage for three different temperatures and different values of Δ*V_G_*. (b) Parameter *β* (symbols) as a function of temperature for three selected gate voltages. The dashed lines correspond to a linear fit. (c) Resistance as a function of temperature measured for different sweep procedures: cooling down (triangle) and warming up (square) at *V*_G_ = 0 (square), warming up with *V*_G_ = 0 after a sweep to *V*_G_ = −200 V (cross) and warming up with *V*_G_ = 0 after a sweep to *V*_G_ = +200 V (circle).

In the limit 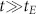, equation (6) reduces to *R*(*t*) = *α* + *β* log *t* where 
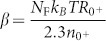
. Figure 8a and 8b show that the *β* parameter increases linearly with temperature, as expected for a thermally activated mechanism. As already mentioned, electrons escaping into the SrTiO_3_ substrate get trapped by the defects of the crystal and no longer contribute to electronic transport. They can be released into the 2-DEG if the temperature of the sample increases above two characteristic values *T*_1_ ≈ 70 K and *T*_2_ ≈ 170 K for our LaTiO_3_/SrTiO_3_ sample (Fig. 8b). The trapping energy inferred from the temperature *T*_1_ is approximately 6 meV. As the electrons are trapped at a typical distance *t* from the interface comparable to the quantum well extension (~10 nm) which is much smaller than the thickness *d* = 500 *μ*m of the SrTiO_3_ substrate, it is not possible to de-trapp the electrons with a negative gate voltage of reasonable value. Indeed, the potential energy transferred the electrons *eV*_G_ × *t*/*d* always remains negligible compare to the trapping one. For this reason we do not observe hysteresis for negative gate voltages.

The same de-trapping behaviour has also been reported in LaAlO_3_/SrTiO_3_^30^ at similar temperatures. The authors associated this behaviour to a thermally activated mechanism supported by exponential relaxations of conductivity near 70 K and 160 K. However, this behaviour should not be confused with the low-temperature logarithmic relaxations observed after a gate voltage step that we described in the present article. The relaxations at 70 K and 160 K are caused by thermal escape of electrons from traps with well-defined energy barriers, giving single exponential relaxations. In contrast, the low-temperature relaxations during the first positive polarisation are caused by electrons escaping from the quantum well with a time-dependent energy barrier, leading to logarithmic time dependence after a characteristic escape time. Similarly, relaxations associated to photoconductivity^32^,^33^ or relaxations sometimes observed at high temperature in SrTiO_3_ based structures and attributed to anions or vacancy diffusion^12^,^31^ also differ from the behaviour discussed in the present article.

In this article, we have taken into account only light electron bands to illustrate the quantum well. Note that the escape mechanism described here is inherent to the presence of a quantum well at the LaXO_3_(X = Al, Ti) SrTiO_3_ interface, disregarding the details of the band structure. It will remain valid even in the presence of a heavy band that has been theoretically predicted^22^,^25^,^27^ and seen by ARPES measurements to a certain extent^38^, since all the sub-bands cross the Fermi level (see Supplementary Figure S4). The escape mechanism does not depend on the exact shape of the potential well, which can be slightly modified by the presence of non-mobile charges at the interfaces (trapped electrons, impurities…) and can vary from sample to sample. It also does not depend strongly on the absolute carrier density of the as-grown sample since the location of the Fermi energy close to the top of quantum well at zero gate voltage is a simple consequence of the Poisson's equation.

In summary, we have shown that in LaAlO_3_/SrTiO_3_ and LaTiO_3_/SrTiO_3_ heterostructures, the Fermi level is instrinsically close to the top of the quantum well after the cool-down. When the carrier density is increased by an electrostatic back-gate voltage beyond a critical value, electrons escape into the SrTiO_3_ substrate at a rate well explained by a thermally activated leakage from the well. This phenomenon which appears both in LaAlO_3_/SrTiO_3_ and LaTiO_3_/SrTiO_3_ heterostructures, is directly responsible for the saturation of the 2-DEG properties with gate voltage, including the mobility and the carrier density, as well as the superconducting transition temperature observed at lower temperature. The exact capacity of the well– and, thus, the maximum carrier density– is mainly determined by growth conditions and can vary from sample to sample. While it is possible to deplete reversibly the quantum well by electrostatic back-gating, the filling is limited by the intrinsic location of the Fermi energy. To overcome this problem we suggest using double gated structures: A back gate could be used to engineer the shape of the quantum well which determines the carrier mobility through the bending of the SrTiO_3_ conduction band. In conjunction, a top gate can be used to add electrons to the well^34^,^35^.

## Methods

### Growth of the heterostructures

LaTiO_3_/SrTiO_3_ hetero-structures were grown at ITT Kanpur (India) using excimer laser based PLD system on commercially available (Crystal GmbH Germany) single crystal substrates of SrTiO_3_ (100) oriented. The substrates were treated with buffered HF to expose TiO_2_ terminated surface. Before deposition, the substrates were heated to 850–950°C for one hour in an oxygen pressure of 200 mTorr to realize surface reconstruction. The source of LaTiO_3_ was a stoichiometric sintered target of 22 mm in diameter which was ablated in an oxygen partial pressure of 1 × 10^−4^ Torr with energy fluence of 1 J/cm^2^ per pulse at a repetition rate of 3 Hz to achieve a growth rate of 0.12 Å/s. Under these conditions, the LaTiO_3_ phase is grown on SrTiO_3_ substrates, as shown by X-Rays diffraction patterns^4^. In this study, we used 15 u.c. thick LaTiO_3_ layers on 0.5 mm thick SrTiO_3_ substrates.

LaAlO_3_/SrTiO_3_ heterostructures were fabricated at UMR CNRS/Thales (Paris, France). A thin LaAlO_3_ film was deposited by PLD (Surface PLD system) on a TiO_2_-terminated (001)-oriented SrTiO_3_ substrate (Crystec and SurfaceNet). A buffered HF treatment followed by annealing, as described in Ref. [36], was used to obtain the TiO_2_ termination required to obtain the conducting electronic system at the interface. The KrF excimer (248 nm) laser ablates the single-crystalline LaAlO_3_ target at 1 Hz, with a fluence between 0.6 and 1.2 J/cm^2^ in an O_2_ pressure of 2 × 10^−4^ mbar. The substrate was typically kept at 730C° during the growth, monitored in real-time by RHEED. As the growth occurs layer-by-layer, it allows us to control the thickness at the unit cell level. After the growth of the film, the sample is cooled down to 500°C in 10^−1^ mbar of O_2_, where the oxygen pressure is increased up to 400 mbar. To reduce the presence of oxygen vacancies (in both the substrate and the film), the sample stays in these conditions for 30 minutes before being cooled down to room temperature^37^. The substrate-target distance was about 57 mm, leading to a growth rate of about 0.2 Å/s in the above conditions. In this study, we used 5 u.c. thick LaAlO_3_ layers on 0.5 mm thick SrTiO_3_ substrates.

## Author Contributions

A.R. and R.C.B. prepared the LaTiO_3_/SrTiO_3_ samples. N.R. and E.L. prepared the LaAlO_3_/SrTiO_3_ samples. J.B. and S.H. performed the measurements, assisted by C.F.-P., J.B., J.L. and N.B. carried out the analysis of the results and wrote the article.

## Supplementary Material

Supplementary Information Supplementary information

## Figures and Tables

**Figure 1:**
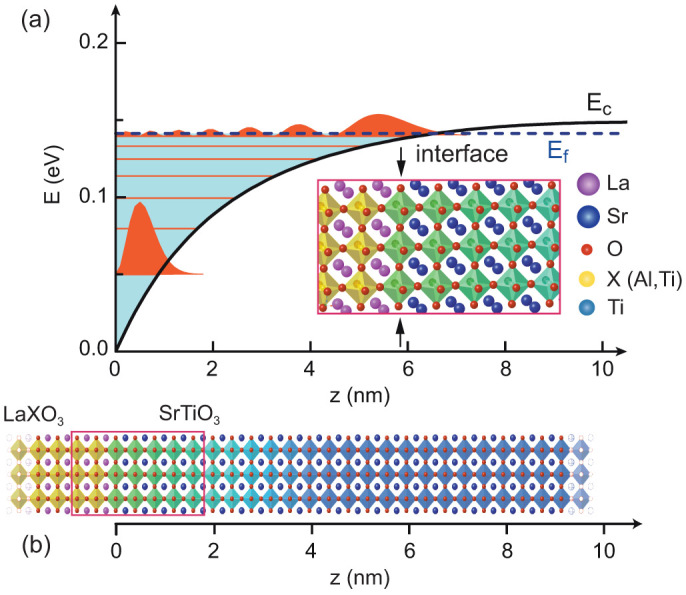
(a) Theoretical calculation of the quantum well profile at an LaXO_3_(X = Al, Ti)/SrTiO_3_ interface for a sample with a carrier density of 7.3 × 10^13^ e^−^/cm^2^. Shown are the SrTiO_3_ conduction band profile *E*_C_ (black), the Fermi energy *E*_F_ (blue dashed line) and the subbands energies (red) as a function of depth *z* from the interface. Only light *d_xy_* electron bands have been taken into account to illustrate the quantum well (see Model and Discussion part for more details). The square modulus of the envelope function of the first and last filled sub-bands are indicated in arbitrary units (red areas). (b) and inset of panel (a): Schematic description of the crystallographic structure at interface.

## References

[b1] Ohtomo A. & Hwang H. Y. A high-mobility electron gas at the *LaAlO*_3_/*SrTiO*_3_ heterointerface. Nature 427, 423–426 (2004). 14749825 10.1038/nature02308

[b2] Reyren N. *et al.* Superconducting interfaces between insulating oxides. Science 317, 1196–1199 (2007). 17673621 10.1126/science.1146006

[b3] Perna P. *et al.* Conducting interfaces between band insulating oxides: The *LaGaO*_3_/*SrTiO*_3_ heterostructure. Appl. Phys. Lett. 97, 152111 (2010).

[b4] Biscaras J. *et al.* Two-dimensional superconductivity at a Mott insulator/band insulator interface *LaTiO*_3_/*SrTiO*_3_. Nature Commun. 1, 89 (2010). 20981013 10.1038/ncomms1084

[b5] Brinkman A. *et al.* Magnetic effects at the interface between non-magnetic oxides. Nature Mater. 6, 493–496 (2007). 17546035 10.1038/nmat1931

[b6] Ben Shalom *et al.* Anisotropic magnetotransport at the *SrTiO*_3_/*LaAlO*_3_ interface. Phys. Rev. B 80, 140403 (2009).

[b7] Li L., Richter C., Mannhart J. & Ashoori R. C. Coexistence of magnetic order and two-dimensional superconductivity at *LaAlO*_3_/*SrTiO*_3_ interfaces. Nature Phys. 7, 762–766 (2011).

[b8] Bert J. A. *et al.* Direct imaging of the coexistence of ferromagnetism and superconductivity at the *LaAlO*_3_/*SrTiO*_3_ interface. Nature Phys. 7, 767–771 (2011).

[b9] Reyren N. *et al.* Anisotropy of the superconducting transport properties of the *LaAlO*_3_/*SrTiO*_3_ interface. Appl. Phys. Lett. 94, 112506 (2009).

[b10] Copie O. *et al.* Towards Two-Dimensional Metallic Behavior at *LaAlO*_3_/*SrTiO*_3_ Interfaces. Phys. Rev. Lett. 102, 216804 (2009). 19519126 10.1103/PhysRevLett.102.216804

[b11] Basletic M. *et al.* Mapping the spatial distribution of charge carriers in *LaAlO*_3_/*SrTiO*_3_ heterostructures. Nature Mat. 7, 621–625 (2008). 10.1038/nmat2223 18587402

[b12] Thiel S., Hammerl G., Schmehl A., Schneider C. W. & Mannhart J. Electron Gases in Oxide Heterostructures. Science 313, 1942–1945 (2006). 16931719 10.1126/science.1131091

[b13] Caviglia A. D. *et al.* Electric field control of the *LaAlO*_3_/*SrTiO*_3_ interface ground state. Nature 456, 624 (2008). 19052624 10.1038/nature07576

[b14] Biscaras J. *et al.* Two-dimensional superconductivity induced by high-mobility carrier doping in *LaTiO*_3_/*SrTiO*_3_ heterostructures. Phys. Rev. Lett. 108, 247004 (2012). 23004312 10.1103/PhysRevLett.108.247004

[b15] Biscaras J. *et al.* Multiple Quantum Criticality in a two-dimensional superconductor. Nature Mat. 12, 542–548 (2013). 10.1038/nmat3624 23584144

[b16] Bibes M. Villegas J. E. & Barthlmy A. Ultrathin oxide films and interfaces for electronics and spintronics. Adv. Phys. 60, 5–84 (2011).

[b17] Takagi H. & Hwang H. Y. An Emergent Change of Phase for Electronics. Science 327, 1601–1602 (2010). 20339063 10.1126/science.1182541

[b18] Mannhart J. & Schlom D. G. Oxide Interfaces - An Opportunity for Electronics. Science 327, 1607–1611 (2010). 20339065 10.1126/science.1181862

[b19] Hwang H. Y. *et al.* Emergent phenomena at oxide interfaces. Nature Mat. 11, 103–113 (2012). 10.1038/nmat3223 22270825

[b20] Bell C. *et al.* Dominant Mobility Modulation by the Electric Field Effect at the *LaAlO*_3_/*SrTiO*_3_ Interface. Phys. Rev. Lett. 103, 226802 (2009). 20366118 10.1103/PhysRevLett.103.226802

[b21] Caviglia A. D. *et al.* Tunable Rashba Spin-Orbit Interaction at Oxide Interfaces. Phys. Rev. Lett. 104, 126803 (2010). 20366557 10.1103/PhysRevLett.104.126803

[b22] Popovic Z., Satpathy S. & Martin R. M. Origin of the Two-Dimensional Electron Gas Carrier Density at the LaAlO_3_ on SrTiO_3_ Interface. Phys. Rev. Lett. 101, 256801 (2008). 19113736 10.1103/PhysRevLett.101.256801

[b23] Kancharla S. S. & Dagotto E. Metallic interface at the boundary between band and Mott insulators. Phys. Rev. B 74, 195427 (2006).

[b24] Salluzzo M. *et al.* Orbital Reconstruction and the Two-Dimensional Electron Gas at the *LaAlO*_3_/*SrTiO*_3_ Interface. Phys. Rev. Lett. 102, 166804 (2009). 19518739 10.1103/PhysRevLett.102.166804

[b25] Son W.-J., Cho E., Lee B., Lee J. & Han S. Density and spatial distribution of charge carriers in the intrinsic n-type *LaAlO*_3_/*SrTiO*_3_ interface. Phys. Rev. B 79, 245411 (2009).

[b26] Delugas P. *et al.* Spontaneous 2-Dimensional Carrier Confinement at the n-Type *SrTiO*_3_/*LaAlO*_3_ Interface. Phys. Rev. Lett. 106, 166807 (2011). 21599400 10.1103/PhysRevLett.106.166807

[b27] Park S. Y. & Millis A. J. Charge density distribution and optical response of the *LaAlO*_3_/*SrTiO*_3_ interface. Phys. Rev. B 87, 205145 (2013).

[b28] Meevasana W. *et al.* Creation and control of a two-dimensional electron liquid at the bare *SrTiO*_3_ surface. Nature Mater. 10, 114–118 (2011). 21240289 10.1038/nmat2943

[b29] Neville R. C., Hoeneisen B. & Mead C. A. Permittivity of Strontium Titanate. J. Appl. Phys. 43, 2124 (1972).

[b30] Seri S., Schultz M. & Klein L. Thermally activated recovery of electrical conductivity in *LaAlO*_3_/*SrTiO*_3_. Phys. Rev. B 87, 125110 (2013).

[b31] Shultz M. & Klein L. Relaxation of transport properties in electron-doped *SrTiO*_3_. Appl. Phys. Lett. 91, 151104 (2007).

[b32] Rastogi A., Pulikkotil J. J., Auluck S., Hossain Z. & Budhani R. C. Photoconducting state and its perturbation by electrostatic fields in oxide-based two-dimensional electron gas. Phys. Rev. B 86, 075127 (2012).

[b33] Di Gennaro E. *et al.* Persistent Photoconductivity in 2D Electron Gases at Different Oxide Interfaces. Adv. Optical Mater. 1, 834–843 (2013).

[b34] Forg B., Richter C. & Mannhart J. Field-effect devices utilizing *LaAlO*_3_ – *SrTiO*_3_ interfaces. Appl. Phys. Lett. 100, 053506 (2012).

[b35] Hosoda M., Hikita Y., Hwang H. Y. & Bell C. Transistor operation and mobility enhancement in top-gated *LaAlO*_3_/*SrTiO*_3_ heterostructures. Appl. Phys. Lett. 103, 103507 (2013).

[b36] Koster G., Kropman B. L., Rijnders G. J. H. M., Blank D. H. A. & Rogalla H. Quasi-ideal strontium titanate crystal surfaces through formation of strontium hydroxide. Appl. Phys. Lett. 73, 2920 (1998).

[b37] Cancellieri C. *et al.* Influence of the growth conditions on the *LaAlO*_3_/*SrTiO*_3_ interface electronic properties. Europhys. Lett. 91, 17004 (2010).

[b38] Berner G. *et al.* Direct k-Space Mapping of the Electronic Structure in an Oxide-Oxide Interface. Phys. Rev. Lett. 110, 247601 (2013). 25165961 10.1103/PhysRevLett.110.247601

