# Supplementary material for: Limit of the electrostatic doping in two-dimensional electron gases of LaXO3(X = Al, Ti)/SrTiO3
Source: Sci Rep. 2014 Oct 27;4:6788. doi: 10.1038/srep06788 (PMC4209450; doi:10.1038/srep06788)
Supplement: Supplementary Information [file srep06788-s1.pdf]

Supplementary Material

Limit of the electrostatic doping in two-dimensional electron gases of $LaXO_3(X = Al, Ti)/SrTiO_3$

J. Biscaras[1], S. Hurand[1], C. Feuillet-Palma[1], A. Rastogi[2], R. C. Budhani[2,3], N. Reyren[4], E. Lesne[4], J. Lesueur[1], N. Bergeal[1]

[1] *LPEM- UMR8213/CNRS - ESPCI ParisTech - UPMC, 10 rue Vauquelin - 75005 Paris, France*

[2] *Condensed Matter - Low Dimensional Systems Laboratory, Department of Physics, Indian Institute of Technology Kanpur, Kanpur 208016, India*

[3] *National Physical Laboratory, New Delhi - 110012, India and*

[4] *Unité Mixte de Physique CNRS-Thales, 1 Av. A. Fresnel, 91767 Palaiseau, France*

I. Evolution of the carrier density and mobility during the first positive polarisation

Measurements performed at high magnetic field (45 T) during the first positive polarisation reveal that the Hall resistance is not linear with magnetic field (Figure S1). Two distinct slopes are evidenced at low and high field respectively, suggesting a multi-band transport scenario. Following the analysis of reference [1] the Hall resistance has been fitted with a two-band model :

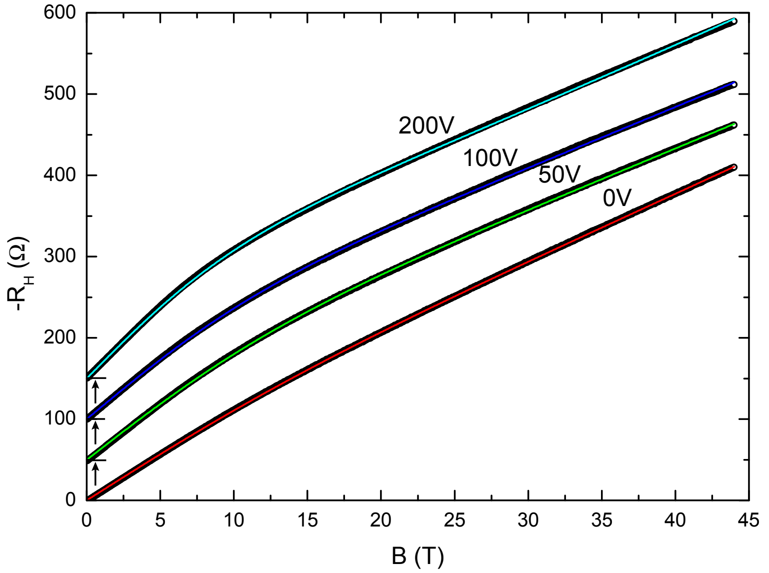

Figure S1: Hall resistance as a function of magnetic field for the first positive polarization measured at 4.2 K on the $LaTiO_3/SrTiO_3$ sample. Open symbols correspond to experimental data, and full lines to fits with expression (1). An offset, indicated by a black horizontal segment and an arrow, has been added to separate the curves.

$$R_{\rm Hall} = \frac{B}{e} \frac{\frac{n_1\mu_1^2}{1+\mu_1^2B^2} + \frac{n_2\mu_2^2}{1+\mu_2^2B^2}}{\left[\frac{n_1\mu_1}{1+\mu_1^2B^2} + \frac{n_2\mu_2}{1+\mu_2^2B^2}\right]^2 + \left[\frac{n_1\mu_1^2B}{1+\mu_1^2B^2} + \frac{n_2\mu_2^2B}{1+\mu_2^2B^2}\right]^2} \quad (1)$$

where n_1 and n_2 are the 2D electron densities and, μ_1 and μ_2 the corresponding mobilities, with the constraint $1/R_s = en_1\mu_1 + en_2\mu_2$. Figure S2 shows the evolution of the different fitting parameters with gate voltage. A majority of low and constant mobility μ_1 carriers (density n_1) is present for all gate biases, whereas a minority of mobile electrons (density n_2) with a mobility μ_2 is present for positive gate voltages only. In reference [1] we proposed that the carriers of density n_1 populating the lowest energy sub-bands are confined close to the interface and have a low-mobility. When the Fermi energy is closed to the top of the well and when a positive gate voltage is applied, we expect carriers of density n_2 to fill the highest energy sub-bands and delocalize deeper in the $SrTiO_3$ substrate where they recover a higher mobility (less scattering and better screening due to a stronger dielectric constant) [1]. During the first positive polarisation, the total carrier density does not change significantly since the Fermi level is already close to the top of the well. However, the conduction band of $SrTiO_3$ bends slightly and some carriers are pulled away from the interface. As a result we inject electrostatically a very small number of highly-mobile carriers (density n_2)

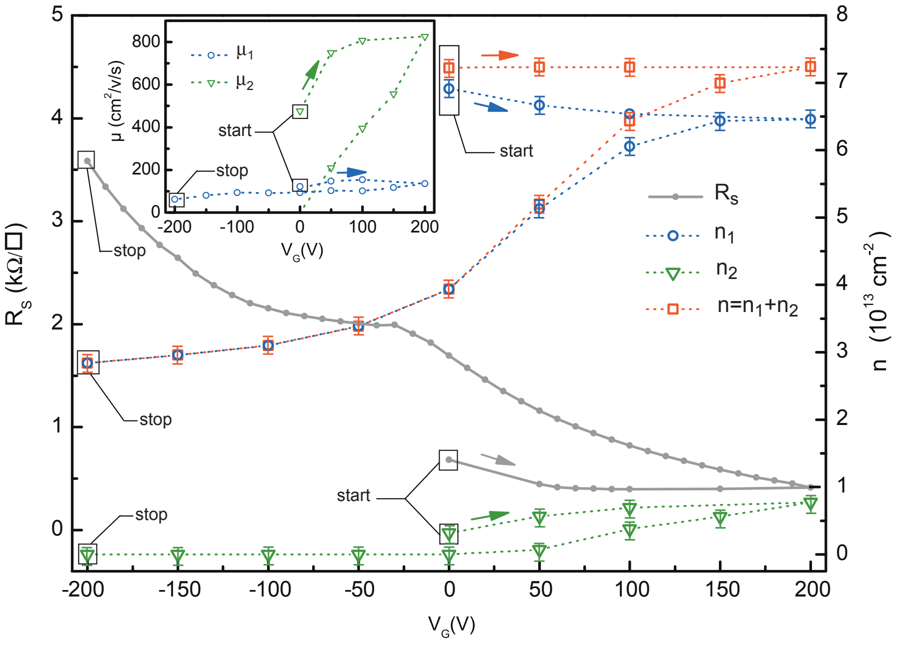

Figure S2: Resistance of the 2-DEG (left scale) and carrier densities n_1, n_2 and n (right scale) as a function of gate voltage measured for the first positive polarisation and backward sweep. The starting points, the stopping points and the directions of the sweeps are indicated on the graph. Inset) Mobilities μ_1 and μ_2 of the two types of carriers measured for the first positive polarisation and backward sweep.

which produce a small decrease of the resistance before reaching saturation. The slight decreases of the density n_1 is compensated by a slight increase of the n_2 one. The inset of figure S2 shows the evolution of the mobilities μ_1 and μ_2 during the first positive polarisation, which is consistent with the behaviour of the carrier densities. Indeed, the mobility μ_1 of the carriers confined deep in the well is found to be rather constant. On the other hand, the mobility μ_2 increases rapidly for positive gate voltage before saturating, indicating that the electron added fills the last subbands accessible before escaping from the well.

II. Characteristic charging time of the sample

The 2-DEG carrier density at the interface can be electrostatically tuned by a metallic gate deposited at the back of the $SrTiO_3$ substrate. The heterostructure forms a capacitor, whose plates consist of the metallic gate on one side and the 2-DEG on the other side, and whose dielectric material is the $SrTiO_3$ substrate. As shown by the scheme in Figure S3, the heterostructure of capacitance C is placed in serial with an external resistor of resistance R_G defining a simple serial

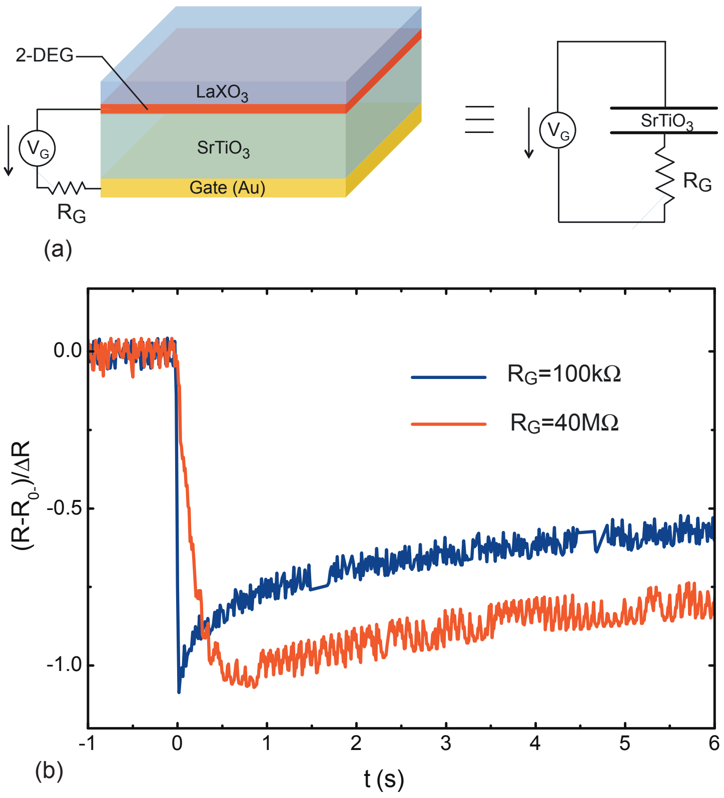

Figure S3: (a) Electrical scheme showing the RC circuit formed by the heterostructure and the external resistance. b) Relaxation of the normalised resistance of the 2DEG after a gate voltage step $\Delta V_{\mathrm{G}} = 10$ V for two different external resistances R_G. The characteristic time $\tau = R_G C$ is un-resolved for R_G=100 kΩ.

RC circuit. In a simple Drude model, the resistance of the 2-DEG after a voltage step reach it's final value with a time dependence $\exp(-t/\tau)$ where $\tau = R_G C$. Figure S3 shows the resistance of the 2-DEG as a function of time, after applying a gate voltage step $\Delta V_{\rm G} = 10$ V, for two different values of the external resistance R_G. The blue curve corresponds to the short $\tau = R_G C$ time (R_G =100 kΩ) used for all the experiments reported in the main text. It shows a sharp decrease of resistivity, followed by the logarithmic relaxation corresponding to electrons escaping from the well. On the other hand, the red curve corresponds to a longer $\tau = R_G C$ time (R_G =40 MΩ) and shows that the initial decrease of the resistance is much slower. However, the logarithmic relaxation remains basically unchanged. We thus see that in our experiment the $\tau = R_G C$ time at which the electrons are added to the 2DEG do not interfere with the thermal escape of electrons into the $SrTiO_3$ substrate.

III. Thermal escape of electrons from the quantum well

We have built a model to account for the relaxation of resistivity in the irreversible regime based on the thermal escape of the electrons from the well. The band bending and sub-bands energies of the $LaTiO_3/SrTiO_3$ interface in the irreversible regime are shown in Figure S4 as an illustration [1]. However, this model is very general and does not depend on the details of the well such as its shape.

We consider a 2D potential well with a finite barrier height E_B and a finite number of parabolic sub-bands of energy E_i (i=1,...,n_L). We assume that electrons at the Fermi level $E_{\rm F}$ can thermally jump over the barrier with a first order kinetics :

$$\frac{{\rm d}n}{{\rm d}t} = -kn \tag{2}$$

where n is the density of electrons in the well and k is the kinetic factor. For thermally activated hoping the latter follows an Arrhénius law : $k = \nu e^{-\frac{\Delta}{k_{\rm B}T}}$ where the activation energy is $\Delta = E_B - E_{\rm F}$, and ν is a characteristic frequency factor. Hence, considering that the density of state of a 2D sub-band is constant and that the Fermi level always stay above the highest sub-band level, the equation on $E_{\rm F}$ is :

$$\frac{{\rm d}E_{\rm F}}{{\rm d}t} = -\nu e^{-\frac{E_B - E_{\rm F}}{k_{\rm B}T}} (E_{\rm F} - \frac{1}{n_L}\sum_{i=1}^{n_L} E_L^i) \tag{3}$$

which is equivalent to a problem with only one level $E_L = \sum_{i=1}^{n_L} E_i/n_L$ but with a density of state multiplied by n_L. Considering low temperatures ($k_B T << E_{\rm F} - E_L$), the variations of $E_{\rm F}$ can be

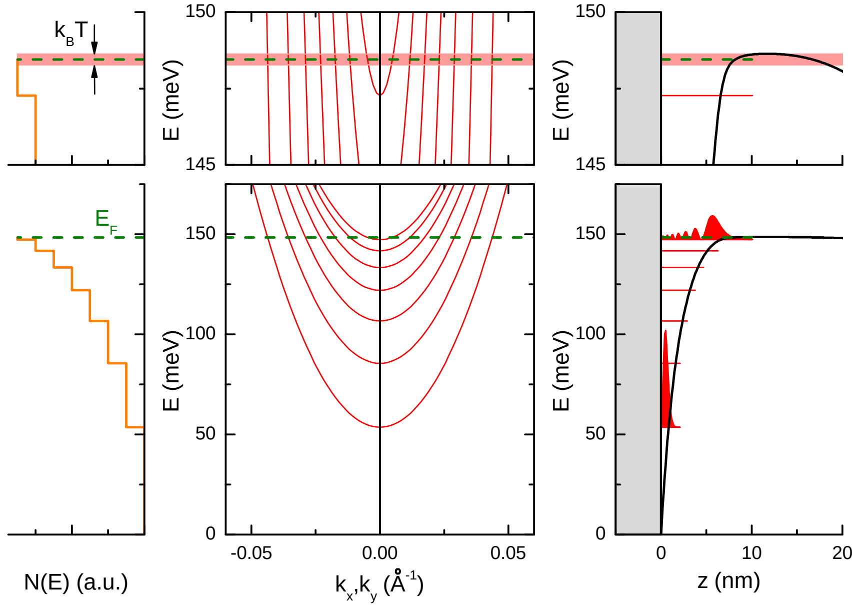

Figure S4: Energy diagram of the quantum well at the $LaTiO_3/SrTiO_3$ interface in the irreversible regime. Left : Density of states in unit of $m/\pi\hbar^2$. Middle : dispersion of the electronic sub-bands in the (k_x , k_y) plane. Right : bending of the conduction band (black) in the z-direction. Energy levels and the first and last envelope function are plotted in red. Down : full energy scale. Top : blow-up near the maximum of the band. The pink horizontal band represent the thermal energy at 4.2 K, centered around the Fermi level (green dashed line).

neglected in $(E_\mathrm{F} - E_L)$ at first order, which gives the solution :

$$E_\mathrm{F}(t) = E_\mathrm{F}^{0^+} - k_B T \ln\left(1 + \frac{t}{t_E}\right) \tag{4}$$

where $E_\mathrm{F}^{0^+}$ is the Fermi level after the voltage step at t=0^+ and t_E is the characteristic escape time :

$$t_E = \frac{k_B T}{\nu(E_\mathrm{F}^{0^+} - E_L)} e^{\frac{E_B - E_\mathrm{F}^{0^+}}{k_\mathrm{B} T}} = \frac{k_B T N_\mathrm{F}}{\nu n_{0^+}} e^{\frac{E_B - E_\mathrm{F}^{0^+}}{k_\mathrm{B} T}} \tag{5}$$

where $N_\mathrm{F} = n_L m/\pi\hbar^2$ is the total density of state at the Fermi level.

It follows that the Fermi level is constant for $t < t_E$ and then decreases logarithmically at longer time. Hence, the conductivity calculated from a simple Drude model with a constant mobility μ is given by

$$\sigma(t) = e\mu\left[n_{0^+} - N_\mathrm{F} k_B T \ln\left(1 + \frac{t}{t_E}\right)\right] \tag{6}$$

At first order we obtain the time evolution of the resistivity

$$R(t) = R_{0^+}\left[1 + \frac{N_\mathrm{F} k_B T}{n_{0^+}} \ln\left(1 + \frac{t}{t_E}\right)\right] \tag{7}$$

where $R_{0^+} = (e\mu n_{0^+})^{-1}$ is the resistivity just after the step. As shown in Figure 7 of the main text and in Supplementary Figure S5, this time dependence of the resistance fits the experimental data with a very good accuracy for both samples.

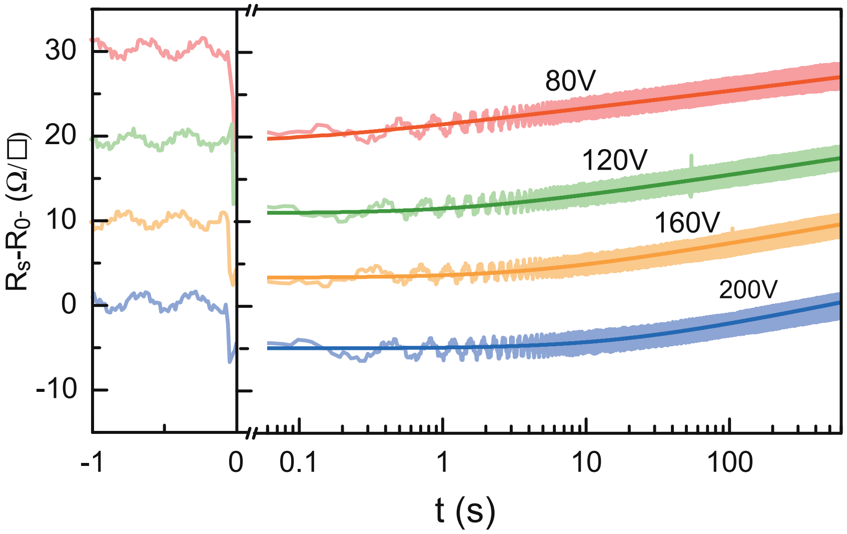

Figure S5: Resistance over time for the $LaAlO_3/SrTiO_3$ sample, measured at 4.2 K for different gate voltages after a ΔV_G=+10 V step, fitted by equation (7).

To analyse the experimental behaviour of the escape time t_E with gate voltage, we now consider a positive gate voltage step ΔV_{G} at $t = 0$. Starting from a density of electrons n_{0^-}, the density after the step is :

$$n_{0^+} = n_{0^-} + \frac{1}{e}\int_{V_{\mathrm{G}}^{0-}}^{V_{\mathrm{G}}^{0+}} C_a(V_{\mathrm{G}})\mathrm{d}V \tag{8}$$

where $C_a(V_{\mathrm{G}})$ is the capacitance per unit area of the sample. For small ΔV_{G}, the variations of C_a with V_{G} can be neglected, and the Fermi level is given by

$$E_{\mathrm{F}}^{0+} = E_{\mathrm{F}}^{0-} + \frac{C_a(V_{\mathrm{G}})\Delta V_{\mathrm{G}}}{eN_{\mathrm{F}}} \tag{9}$$

When replaced in equation (5), the variation of t_E with C_a and ΔV_{G} is given by

$$\ln(t_E) = \gamma - \kappa C_a(V_{\mathrm{G}})\Delta V_{\mathrm{G}} \tag{10}$$

where :

$$\gamma = \ln\left(\frac{N_{\mathrm{F}}k_BT}{\nu n_{0+}}\right) + \frac{E_B - E_{\mathrm{F}}^{0-}}{k_BT}$$

$$\kappa = \frac{1}{eN_{\mathrm{F}}k_BT}$$

In the irreversible regime, the variations of $\ln(t_E)$ follow a linear dependence with both C_a and ΔV_{G}, as shown in Figure 7 of the main text.

[1] J. Biscaras et al. Phys. Rev. Lett. **108**, 247004 (2012).
[2] R. Neville et al. J. Appl. Phys. **43**, 2124–2131 (1972).
[3] J. Biscaras *et al.*, Nature Communications **1**, 89 (2010).
[4] G. Koster et al. Appl. Phys. Lett.,**73**, 2920 (1998).
[5] C. Canciellieri, Europhys. Lett. **91**, 1704 (2010).
